# Evaluation of the Eclipse eMC algorithm for bolus electron conformal therapy using a standard verification dataset

**DOI:** 10.1120/jacmp.v17i3.5885

**Published:** 2016-05-08

**Authors:** Robert L. Carver, Conrad P. Sprunger, Kenneth R. Hogstrom, Richard A. Popple, John A. Antolak

**Affiliations:** ^1^ Mary Bird Perkins Cancer Center Baton Rouge LA USA; ^2^ Department of Physics and Astronomy Louisiana State University Baton Rouge LA USA; ^3^ Department of Radiation Oncology The University of Alabama at Birmingham Birmingham AL USA; ^4^ Department of Radiation Oncology Mayo Clinic Rochester MN USA

**Keywords:** bolus, electron therapy, conformal therapy, electron Monte Carlo

## Abstract

The purpose of this study was to evaluate the accuracy and calculation speed of electron dose distributions calculated by the Eclipse electron Monte Carlo (eMC) algorithm for use with bolus electron conformal therapy (ECT). The recent commercial availability of bolus ECT technology requires further validation of the eMC dose calculation algorithm. eMC‐calculated electron dose distributions for bolus ECT have been compared to previously measured TLD‐dose points throughout patient‐based cylindrical phantoms (retromolar trigone and nose), whose axial cross sections were based on the mid‐PTV (planning treatment volume) CT anatomy. The phantoms consisted of SR4 muscle substitute, SR4 bone substitute, and air. The treatment plans were imported into the Eclipse treatment planning system, and electron dose distributions calculated using 1% and <0.2% statistical uncertainties. The accuracy of the dose calculations using moderate smoothing and no smoothing were evaluated. Dose differences (eMC‐calculated less measured dose) were evaluated in terms of absolute dose difference, where 100% equals the given dose, as well as distance to agreement (DTA). Dose calculations were also evaluated for calculation speed. Results from the eMC for the retromolar trigone phantom using 1% statistical uncertainty without smoothing showed calculated dose at 89% (41/46) of the measured TLD‐dose points was within 3% dose difference or 3 mm DTA of the measured value. The average dose difference was −0.21%, and the net standard deviation was 2.32%. Differences as large as 3.7% occurred immediately distal to the mandible bone. Results for the nose phantom, using 1% statistical uncertainty without smoothing, showed calculated dose at 93% (53/57) of the measured TLD‐dose points within 3% dose difference or 3 mm DTA. The average dose difference was 1.08%, and the net standard deviation was 3.17%. Differences as large as 10% occurred lateral to the nasal air cavities. Including smoothing had insignificant effects on the accuracy of the retromolar trigone phantom calculations, but reduced the accuracy of the nose phantom calculations in the high‐gradient dose areas. Dose calculation times with 1% statistical uncertainty for the retromolar trigone and nose treatment plans were 30 s and 24 s, respectively, using 16 processors (Intel Xeon E5‐2690, 2.9 GHz) on a framework agent server (FAS). In comparison, the eMC was significantly more accurate than the pencil beam algorithm (PBA). The eMC has comparable accuracy to the pencil beam redefinition algorithm (PBRA) used for bolus ECT planning and has acceptably low dose calculation times. The eMC accuracy decreased when smoothing was used in high‐gradient dose regions. The eMC accuracy was consistent with that previously reported for accuracy of the eMC electron dose algorithm and shows that the algorithm is suitable for clinical implementation of bolus ECT.

PACS number(s): 87.55.kd

## I. INTRODUCTION

Electron conformal therapy (ECT) aims to match the distal 90% dose surface to the distal surface of the planning target volume (PTV) and to reduce dose to distal healthy tissue. ECT can be achieved by multiple technologies, as reviewed by Hogstrom et al.^(1)^ One of these methods, bolus ECT, uses a variable‐thickness wax bolus placed on the patient surface to conform the distal 90% dose surface to the PTV. Bolus ECT has been shown useful for multiple sites in the head and neck, postmastectomy chest wall, and paraspinal muscles.^2^, ^3^, ^4^, ^5^, ^6^, ^7^, ^8^

Bolus ECT dose conformity to the PTV requires an accurate dose algorithm in the treatment planning system (TPS). Carver et al.^(9)^ measured electron dose distributions for bolus ECT plans on cylindrical patient‐based phantoms of the retromolar trigone and nose.^(10)^ These measurements were used to evaluate the accuracy of the pencil beam algorithm (PBA) in the Pinnacle^3^ TPS (Philips Healthcare, Andover, MD) and the pencil beam redefinition algorithm (PBRA) in the p.d software (.decimal, LLC, Sanford, FL) used for designing bolus ECT. In addition to the PBA and the PBRA, Monte Carlo (MC) algorithms are expected to provide sufficient accuracy for bolus ECT treatment planning.

The Eclipse TPS (Varian Medical Systems, Palo Alto, CA) currently employs the electron Monte Carlo (eMC) algorithm for electron dose calculations. The eMC algorithm is a fast implementation of the Macro Monte Carlo method (MMC) and relies on precalculated data to greatly reduce the calculation time as compared to traditional Monte Carlo methods.^(11)^ Popple et al.^(12)^ showed that the eMC algorithm produces dose distributions that agree well with a standard dataset measured by Boyd et al.^(13)^ That study showed that, in the presence of internal heterogeneities, the eMC matched the measured dataset to within 3% or 3 mm. However, this evaluation did not include data measured in the presence of conformal bolus. With the recent commercial availability of bolus ECT planning and delivery technology (.decimal, LLC, Sanford, FL), the accuracy and computational speed of the eMC algorithm in the presence of conformal bolus is of clinical interest. The purpose of this study was to evaluate the accuracy and calculation speed of the eMC electron dose algorithm found in the Eclipse TPS for bolus ECT plans of a retromolar trigone and nose phantom using the measured data set found in Carver et al.^(9)^ This study uses a 3% or 3 mm accuracy test, as used by Popple et al.,^(12)^ as the benchmark for clinically acceptable algorithm accuracy.

## II. MATERIALS AND METHODS

### A. Eclipse electron Monte Carlo algorithm commissioning

In order to commission an electron beam for Monte Carlo dose calculations, Eclipse required three measured beam‐specific inputs: a depth‐dose curve for the open field (i.e., no applicator, wide open jaws), depth‐dose curve for each applicator used, and an in‐air profile at 95 cm SSD for the open field. These measurements were necessary for the initial phase space (IPS) model to properly model the linear accelerator and its treatment head.

Eclipse eMC commissioning data were measured at Mary Bird Perkins Cancer Center (MBPCC) for a 16 MeV ($E_{p,0}=16.6\;\text{MeV}$) beam on a Clinac EX (Varian Medical Systems) for the $15\times 15\;{\text{cm}}^{2}$ and $10\times 10\;{\text{cm}}^{2}$ applicators, the same linear accelerator Carver et al.^(9)^ used for the standard measured dataset. Beam scanning was performed using a RFA‐200 Water Phantom 2D scanning tank and OmniPro scanning software (IBA Dosimetry, Bartlett, TN). Dose measurements were taken with a N30013 ion chamber (CNMC Company, Nashville, TN).

The resulting commissioned beams were evaluated by comparing calculated with measured central‐axis percent depth‐dose curves and 2D isodose distributions in water. The measured and commissioned beams agreed to within 2%/1 mm. Results shown in this paper reflect dose calculations done at the Mayo Clinic, using 16 processors (Intel Xeon E5‐2690, 2.9 GHz) on a framework agent server (FAS).

### B. Measured dataset description

**Figure 1:**
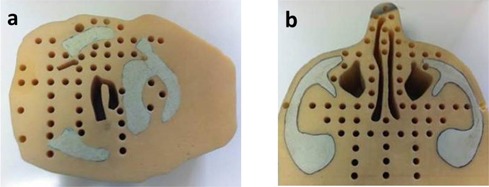
Cylindrical phantoms (height perpendicular to axial plane=7 cm) from Hogstrom et al.^(10)^ were composed of bone and muscle tissue substitutes to model the central CT transverse plane of patients with (a) retromolar trigone and (b) nose cancer. The 2D phantoms have a constant axial cross section with the exception of the holes drilled to house plastic TLD capsules or water. Figure is from Carver et al.^(9)^

Electron dose distributions calculated using the eMC algorithm were compared to a publicly‐available measured dataset consisting of multiple point‐dose measurements for two cylindrical patient‐like phantoms in the presence of conformal bolus.^(9)^ The two cylindrical phantoms used in this study were the same ones Hogstrom et al.^(10)^ used to evaluate the accuracy of the first commercial implementation of the PBA for patients previously treated for a retromolar trigone and nose cancer. The axial cross section of each phantom was based on the mid‐PTV CT scan of a patient. The phantoms, constructed of SR4 muscle substitute, SR4 bone substitute, and air cavities, allowed insertion of TLD capsules for dose measurement (Fig. 1). Recently, data consisting of 46 measured point doses for the retromolar phantom and 57 for the nose phantom were measured by Carver et al.^(9)^ to assess the accuracy of the PBA and PBRA for bolus ECT.

### C. Treatment planning

The treatment plans developed in Eclipse were those described in Carver et al.^(9)^ The retromolar trigone phantom was planned using a 16 MeV beam ($E_{p,0}=16.6\;\text{MeV}$) on a Clinac EX (Varian Medical Systems) at 105 cm SSD with a $7.8\times 11.8\;{\text{cm}}^{2}$ (defined at isocenter) Cerrobend insert in the $15\times 15\;{\text{cm}}^{2}$ applicator, where the 11.8 cm defined the field width in the plane of measurement. The nose phantom was planned using the same energy at 100 cm SSD with an $8.0\times 9.8\;{\text{cm}}^{2}$ (defined at isocenter) Cerrobend insert in the $10\times 10\;{\text{cm}}^{2}$ applicator, where the 9.8 cm defined the field width in the plane of measurement. In both cases the SSD was the central‐axis distance from a point 100 cm above isocenter to the proximal bolus surface.

### D. Dose calculations

The eMC was evaluated for three dose‐calculation parameter sets. The maximum number of histories $\left(2\times 10^{9}\right)$ was used to achieve the lowest possible statistical uncertainty (<0.2%) without smoothing and a 2 mm calculation grid size. This parameter set will be referred to as the max histories setup. We evaluated the algorithm's accuracy with a typical clinical statistical uncertainty of 1%, without smoothing, and a 2 mm calculation grid size. This parameter set will be referred to as the 1% setup. The final setup added 3D smoothing to the 1% setup, which would be typical of many clinical calculations. This parameter set will be referred to as the clinical setup.

The eMC uses precalculated probability densities functions to increase the computational speed compared to traditional MC algorithms. These precalculated probability distribution functions include only five materials (air, lung, water, lucite, and solid bone).^(11)^ If a calculation voxel has a density other than one of the precalculated materials, then the material is randomly selected from the two closest materials for each history. The probability for a precalculated material to be selected is proportional to the closeness of a voxel's average mass density to the mass density of the precalculated material.^(14)^ Since the phantoms and the bolus are not comprised of these five materials, this material approximation represents a possible source of error. The CT data from Carver et al.^(9)^ was imported into Eclipse for dose calculations. Eclipse converts CT number to mass density with scanner‐specific calibration tables.^(14)^

Dose calculations were normalized such that 100% equals the given dose, where the given dose is defined as the maximum central‐axis dose at the treatment SSD in water for the same rectangular field. This is the same normalization used in Carver et al.^(9)^ Differences between eMC‐calculated and measured dose distributions were evaluated in terms of absolute dose difference, as well as distance to agreement (DTA). Dose calculations times were also evaluated to determine the clinical acceptability for use with bolus ECT.

## III. RESULTS

### A. Accuracy of eMC for retromolar trigone phantom with bolus

**Figure 2:**
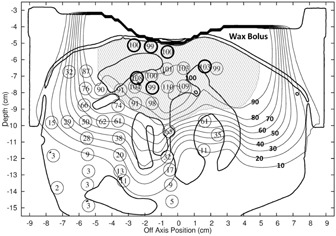
Measured TLD doses (N=46) for the retromolar trigone phantom with conformal bolus (16 MeV) superimposed on isodose plot of eMC‐calculated dose distribution for the clinical setup. Isodose lines, labeled in bold, are % of given dose. The shaded region represents the physician‐delineated PTV; the narrow band between the phantom and bolus is a small air gap. Points having a dose difference >3% and DTA>3 mm are indicated by bold circles.

**Figure 3:**
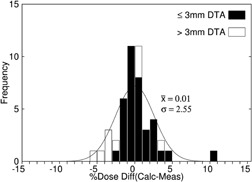
Dose difference histogram DTA>3 mm from data in Fig. 2 for the retromolar trigone phantom with conformal bolus (clinical setup). The parameters of the superimposed Gaussian curve are the average and standard deviation of the measured dose differences. Points with a distance‐to‐agreement (DTA) less than or equal to (greater than) 3 mm are histogrammed in black (white).

For the max histories setup, there was an average dose difference (eMC ‐ measured) of −0.12% and a standard deviation (SD) of 2.56%. This standard deviation is significant compared to 0.9%, the reported average standard error for the mean TLD‐measured dose values, and root‐mean‐square (rms) subtraction of this error resulted in a net standard deviation of 2.40%. For the 1% setup, there was an average dose difference of −0.21% and a standard deviation of 2.49%. An rms subtraction of the TLD measurement error resulted in a net standard deviation of 2.32%. For the clinical setup, there was an average dose difference of 0.01% and a standard deviation of 2.55%. An rms subtraction of the TLD measurement error resulted in a net standard deviation of 2.38%. Differences as large as 3.7% occurred immediately distal to the mandible bone for the clinical setup. Figure 2 shows the measured dose points superimposed on the eMC isodose lines calculated using the clinical setup for the retromolar phantom treatment plan with conformal bolus. Figure 3 shows the histogram of the dose differences, for these data, as well as whether points have a DTA greater than or a DTA less than or equal to 3 mm. Table 1 summarizes the accuracy of the dose calculations and calculation times for the three eMC calculation parameter sets.

**Table 1:** Accuracy of the eMC algorithm vs. dose calculation parameters for the retromolar trigone phantom with conformal bolus. Listed are the approximate calculation time, the number of data points that lie within 3% dose difference or 3 mm DTA, and the average dose difference ±1 standard deviation (SD). For comparison, the average dose difference ±1 SD for the PBA and PBRA have been included.^(9)^

	*Max Histories Setup*	*1% Setup*	*Clinical Setup*
Statistical Uncertainty	<0.2%	1.0%	1.0%
Dose Smoothing	N	N	Y
eMC Calculation Time	15 m	30 s	30 s
eMC ≤3% or 3 mm DTA	41/46 (89%)	41/46 (89%)	40/46 (87%)
eMC Avg. Dose Difference ±1 Net SD	−0.12%±2.40%	−0.21%±2.32%	0.01%±2.38%
PBRA Avg. Dose Difference ±1 Net SD	——–	——–	−0.20%±1.54% ^(9)^
PBA Avg. Dose Difference ±1 Net SD	——–	——–	−0.05%±3.14% ^(9)^

### B. Accuracy of eMC for nose phantom with bolus

**Figure 4:**
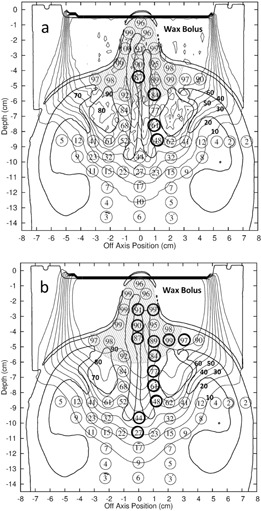
Measured TLD doses (N=57) for the nose phantom with conformal bolus (16 MeV) superimposed on isodose plot of eMC‐calculated dose distribution for the (a) 1% setup and (b) clinical setup. Isodose lines, labeled in bold, are % of the given dose. The shaded region represents the physician‐delineated PTV; the narrow band between the phantom and bolus is a small air gap. Points having a dose difference >3% and a DTA>3 mm are indicated by bold circles.

**Figure 5:**
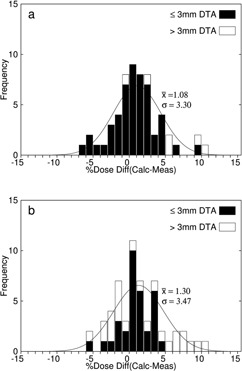
Dose difference histogram (N=57) from data in Fig. 4 for the nose phantom with conformal bolus. The parameters of the superimposed Gaussian curve are the average and standard deviation of the measured dose differences for the (a) 1% setup and (b) clinical setup. Points with a distance‐to‐agreement (DTA) less than or equal to (greater than) 3 mm are histogrammed in black (white).

For the max histories setup, there was an average dose difference (eMC ‐ measured) of 1.12% with a standard deviation of 3.03% and root‐mean‐square (rms) subtraction of the average standard error for the mean TLD measured dose values resulted in a net standard deviation of 2.89%. For the 1% setup, there was an average dose difference of 1.08% with a standard deviation of 3.30%. An rms subtraction of the TLD measurement error resulted in a net standard deviation of 3.17%. For the clinical setup, there was an average dose difference of 1.30% with a standard deviation of 3.47%. An rms subtraction of the TLD measurement error resulted in a net standard deviation of 3.35%. Differences as large as 10.0% occurred lateral to the nasal air cavities for the clinical setup. Figure 4 shows the measured dose points superimposed on the eMC calculation isodose lines for the nose phantom treatment plan with bolus for the 1% setup and clinical setup. Figure 5 shows the histogram of the dose differences for these setups, as well as whether points have a DTA greater than or a DTA less than or equal to 3 mm. Table 2 summarizes the accuracy of the dose calculations and calculation times for the three eMC calculation parameter sets.

**Table 2:** Accuracy of the eMC algorithm vs. dose calculation parameters for the nose with conformal bolus. Listed are the approximate calculation time, the number of data points that lie within 3% dose difference or 3 mm DTA, and the average dose difference ±1 SD. For comparison, the average dose difference ±1 SD for the PBA and PBRA have been included.^(9)^

	*Max Histories Setup*	*1% Setup*	*Clinical Setup*
Statistical Uncertainty	<0.2%	1.0%	1.0%
Dose Smoothing	N	N	Y
eMC Calculation Time	22 m	24 s	24 s
eMC ≤3% or 3 mm DTA	53/57 (93%)	53/57 (93%)	44/57 (77%)
eMC Avg. Dose Difference ±1 Net SD	1.12%±2.89%	1.08%±3.17%	1.30%±3.35%
PBRA Avg. Dose Difference ±1 Net SD	——–	——–	−0.18%±1.22% ^(9)^
PBA Avg. Dose Difference ±1 Net SD	——–	——–	−1.75%±5.94% ^(9)^

## IV. DISCUSSION

The 1% setup produced dose distributions with a net standard deviation of 2.32% and 3.17% for the retromolar trigone and nose bolus cases, respectively, resulting in 89% and 93%, respectively, of points within 3% or 3 mm. Increasing the number of histories for the max histories setup had an insignificant effect on the overall accuracy of the calculation (i.e., same number of dose points passed the 3%/3 mm criteria) but greatly increased the calculation time from 0.5 to 15 min and from 0.4 to 22 min for the retromolar trigone and nose cases, respectively. For both the retromolar trigone and nose bolus cases, a 1% statistical uncertainty achieved clinically acceptable results in a clinically acceptable time frame (<1 min).

For the nose bolus case, there was a significant decrease in the number of calculation points within 3% or 3 mm DTA for the clinical setup (77%) as compared to the 1% setup (93%). However, for the retromolar trigone case, these two setups produced nearly identical results. The nose bolus case had long vertical nasal air cavities that produced nearby high‐gradient dose regions, and the smoothing reduced this gradient and the accuracy of the simulation. The retromolar trigone phantom doesn't contain these types of heterogeneities, thus the effect is not seen. Hence, these dose comparisons illustrate the need to utilize smoothing with care, as smoothing in the vicinity of high dose gradient regions can reduce accuracy, as illustrated for the nose in Table 2.

Results of the eMC algorithm comparisons showed increased accuracy (smaller standard deviation of calculated minus measured dose) as compared to those of the Pinnacle^3^ PBA (1, 2). This result was more significant for the nose case, apparently due to the vertical air heterogeneities, which are a well‐documented difficulty for the PBA.^9^, ^10^ Contrastingly, the eMC algorithm comparison showed slightly decreased accuracy as compared to that of the p.d PBRA, which is used for dose calculations in the bolus creation software p.d, distributed by .decimal, LLC. (http://products.dotdecimal.com/ContentSelection.html?type=Planning)

The dose calculation times for the eMC are comparable with those found for other dose calculation algorithms (i.e., PBA and PBRA). Table 3 shows a comparison of these calculation times, where the standard clinical setup is used for the eMC.

The retromolar and nose bolus cases did not show any significant increase in eMC accuracy when increasing the number of histories beyond what is needed for 1% statistical uncertainty to <0.2% (max histories setup). This trend suggests that the small differences remaining between the measured and calculated dose distributions are caused by nonstatistical errors. Understanding why the eMC was slightly less accurate than the PBRA requires further study to identify the sources of these small errors.

Popple et al.^(12)^ showed that the eMC with 1% statistical uncertainty and without smoothing matched a standard 20 MeV measured dataset with heterogeneities within 3% or 3 mm for 97% of points. Smoothing increased this pass rate to >99%. However, they also showed that smoothing in high‐dose gradient regions reduced accuracy there. The results of this study showed that the eMC without smoothing passed the 3% or 3 mm criteria for 89% and 93% of points for the retromolar trigone and nose bolus cases, respectively. This study also similarly showed that smoothing in high‐gradient dose regions reduced the dose calculation accuracy.

**Table 3:** Comparison of calculation times between the PBA, PBRA, and eMC algorithms. Times shown for the eMC (clinical) refer to 1% statistical uncertainty followed by smoothing.

*Dose Algorithm*	*TPS*	*Processors*	*Retromolar*	*Nose*
PBA	Phillips Pinnacle	1 (Xeon E5‐2690)	<10 s	<10s
PBRA	.decimal p.d	1 (Intel E7500)	45 s	40 s
eMC (clinical)	Varian Eclipse	16 (Xeon E5‐2690)	30 s	24 s

## V. CONCLUSIONS AND RECOMMENDATIONS

Results of this study with bolus were consistent with those previously reported for accuracy of the eMC electron dose algorithm without bolus. However, adding histories to achieve maximum statistical accuracy had an insignificant effect on overall accuracy. Our investigation showed that for bolus ECT cases with high‐gradient dose regions, such as those found lateral to the nasal air cavities of the nose, smoothing the dose calculations can significantly reduce its accuracy. Hence, the eMC dose algorithm is most suitable for clinical implementation of bolus ECT using 1% statistical uncertainty without smoothing. It has comparable accuracy to the PBRA, which is used for conformal bolus design, and has acceptably low dose calculation times.

## COPYRIGHT

This work is licensed under a Creative Commons Attribution 4.0 International License.
